# Supplementary material for: Predicting visual function by interpreting a neuronal wiring diagram
Source: Nature. 2024 Oct 2;634(8032):113–23. doi: 10.1038/s41586-024-07953-5 (PMC11446822; doi:10.1038/s41586-024-07953-5)
Supplement: Supplementary file 6 — CRF and ERF predictions for individual TmY4 and TmY9 cells. Analogous to Supplementary Data 3, but for TmY target types. Shown are the top four monosynaptic pathways, the strongest pathway passing through each of the top ten intermediary types (ranking from Extended Data Fig. 7), and the trisynaptic pathway Tm1–TmY–Dm3–TmY (see the section entitled Prediction of spatial normalization). [file 41586_2024_7953_MOESM6_ESM.zip › DataS4/TmY9q/720575940623294249.pdf]

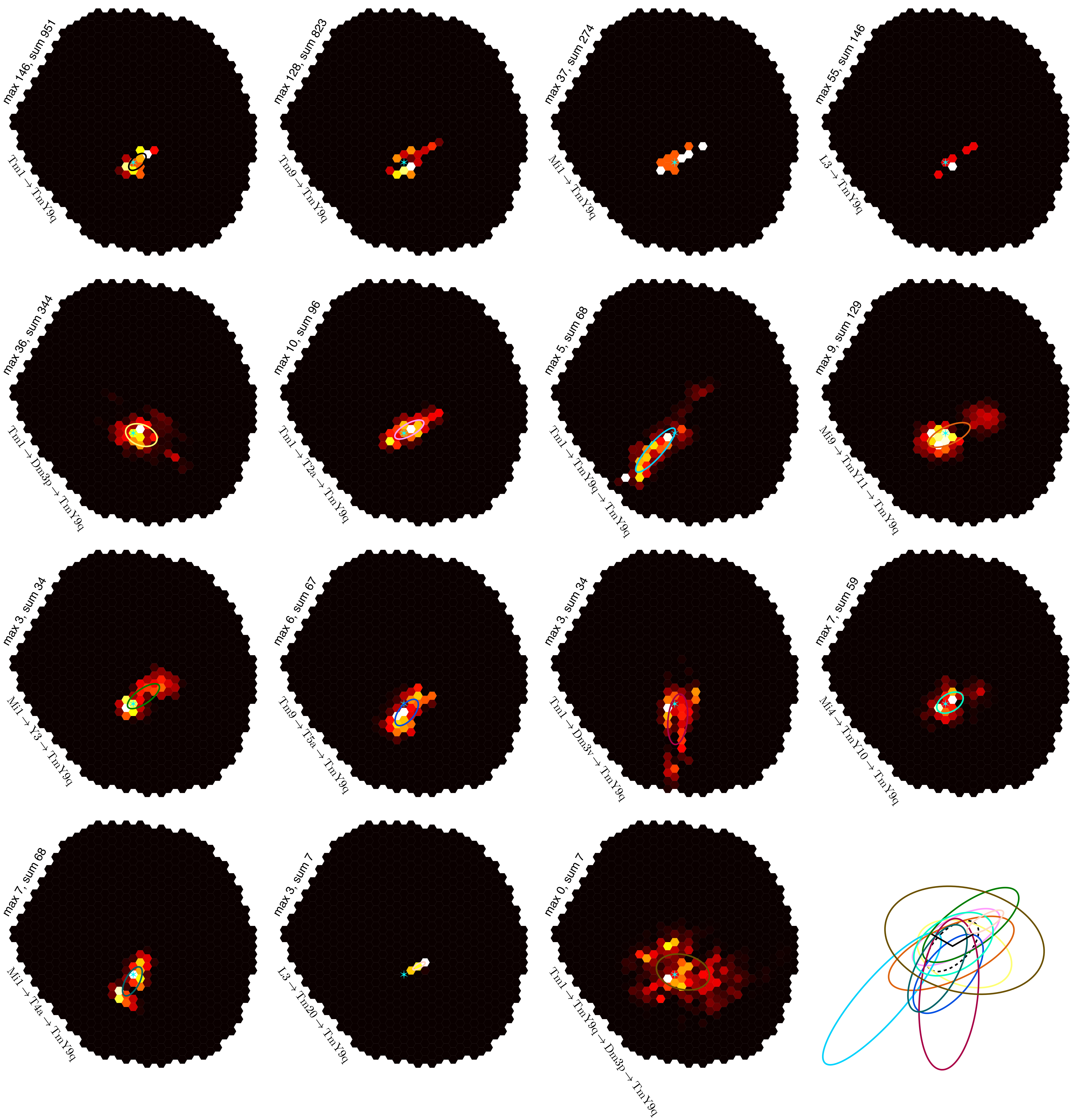

max 146, sum 951
Tm1 → TmY9q
max 128, sum 823
Tm9 → TmY9q
max 37, sum 274
Mi1 → TmY9q
max 55, sum 146
L3 → TmY9q
max 36, sum 344
Tm1 → Dm3p → TmY9q
max 10, sum 96
Tm1 → T2a → TmY9q
max 5, sum 68
Tm1 → TmY9q → TmY9q
max 9, sum 129
Mi9 → TmY11 → TmY9q
max 3, sum 34
Mi1 → Y3 → TmY9q
max 6, sum 67
Tm9 → T5a → TmY9q
max 3, sum 34
Tm1 → Dm3v → TmY9q
max 7, sum 59
Mi4 → TmY10 → TmY9q
max 7, sum 68
Mi1 → T4a → TmY9q
max 3, sum 7
L3 → Tm20 → TmY9q
max 0, sum 7
Tm1 → TmY9q → Dm3p → TmY9q
